# Supplementary material for: Depletion of slow-cycling PDGFRα+ADAM12+ mesenchymal cells promotes antitumor immunity by restricting macrophage efferocytosis
Source: Nat Immunol. 2023 Oct 5;24(11):1867–78. doi: 10.1038/s41590-023-01642-7 (PMC10602852; doi:10.1038/s41590-023-01642-7)
Supplement: Supplementary file 1 — Reporting Summary [file 41590_2023_1642_MOESM1_ESM.pdf]

nature portfolio

Corresponding author(s): Lucie PEDUTO

Last updated by author(s): September 5th, 2023

Reporting Summary

Nature Portfolio wishes to improve the reproducibility of the work that we publish. This form provides structure for consistency and transparency in reporting. For further information on Nature Portfolio policies, see our Editorial Policies and the Editorial Policy Checklist.

Statistics

For all statistical analyses, confirm that the following items are present in the figure legend, table legend, main text, or Methods section.

n/a	Confirmed	
☐	☒	The exact sample size (*n*) for each experimental group/condition, given as a discrete number and unit of measurement
☐	☒	A statement on whether measurements were taken from distinct samples or whether the same sample was measured repeatedly
☐	☒	The statistical test(s) used AND whether they are one- or two-sided<br>*Only common tests should be described solely by name; describe more complex techniques in the Methods section.*
☒	☐	A description of all covariates tested
☐	☒	A description of any assumptions or corrections, such as tests of normality and adjustment for multiple comparisons
☐	☒	A full description of the statistical parameters including central tendency (e.g. means) or other basic estimates (e.g. regression coefficient) AND variation (e.g. standard deviation) or associated estimates of uncertainty (e.g. confidence intervals)
☐	☒	For null hypothesis testing, the test statistic (e.g. *F*, *t*, *r*) with confidence intervals, effect sizes, degrees of freedom and *P* value noted<br>*Give P values as exact values whenever suitable.*
☒	☐	For Bayesian analysis, information on the choice of priors and Markov chain Monte Carlo settings
☒	☐	For hierarchical and complex designs, identification of the appropriate level for tests and full reporting of outcomes
☒	☐	Estimates of effect sizes (e.g. Cohen's *d*, Pearson's *r*), indicating how they were calculated

Our web collection on statistics for biologists contains articles on many of the points above.

Software and code

Policy information about availability of computer code

Data collection	This study does not report original code.
Data analysis	Data acquisition and analysis was performed using the following softwares, as detailed in methods: FlowJo v.10; BD FACSDiva software; ZEN 2 (Zeiss); ImageJ v1.54f; GraphPad Prism 9; Bio-Rad CFX manager 3.1; 2100 Bioanalyzer Expert Software (Agilent); Sequana 0.11.0; Snakemake 6.1.1.

For manuscripts utilizing custom algorithms or software that are central to the research but not yet described in published literature, software must be made available to editors and reviewers. We strongly encourage code deposition in a community repository (e.g. GitHub). See the Nature Portfolio guidelines for submitting code & software for further information.

Data

Policy information about availability of data

All manuscripts must include a data availability statement. This statement should provide the following information, where applicable:

- Accession codes, unique identifiers, or web links for publicly available datasets
- A description of any restrictions on data availability
- For clinical datasets or third party data, please ensure that the statement adheres to our policy

The TCGA_PAAD, TCGA_SKCM, TCGA_COAD and TCGA_PRAD datset can be accessed at https://portal.gdc.cancer.gov/. The ICGC_PAN_AU dataset can be accessed at: https://dcc.icgc.org/. scRNAseq data of pancreatic cancer from Peng et al. (Cell Research 2019) are available from the Genome Sequence Archive (project

PRJCA001063). scRNAseq data from CRC (Pelka et al., Cell 2021) and SKCM (Jerby-Arnon et al., Cell 2018) are accessible via the Gene Expression Omnibus (GEO) under accession no. GSE178341 and GSE115978, respectively. The genome GRCm38 (GCA_000001635.9) release 102 is accessible from Ensembl (http://www.ensembl.org/Mus_musculus/Info/Index). RNA-seq data generated by this study have been deposited in NCBI's Gene Expression Omnibus and are accessible through GEO Series accession number RNA-seq data generated in this study are deposited in NCBI's Gene Expression Omnibus under accession no. GSE206794 and GSE206795.

Human research participants

Policy information about studies involving human research participants and Sex and Gender in Research.

Reporting on sex and gender	N/A
Population characteristics	N/A
Recruitment	N/A
Ethics oversight	N/A

Note that full information on the approval of the study protocol must also be provided in the manuscript.

Field-specific reporting

Please select the one below that is the best fit for your research. If you are not sure, read the appropriate sections before making your selection.

☒ Life sciences ☐ Behavioural & social sciences ☐ Ecological, evolutionary & environmental sciences

For a reference copy of the document with all sections, see nature.com/documents/nr-reporting-summary-flat.pdf

Life sciences study design

All studies must disclose on these points even when the disclosure is negative.

Sample size	No statistical method was used to predetermine sample size. Sample size was chosen based on prior experience and prior published studies with similar layout.
Data exclusions	No data was excluded from the analysis.
Replication	The number of replicates for each experiment is indicated in figure legends. Within each experimental group the reproducibility was successful, although a degree of variability was detected due to inter- individual diversity.
Randomization	Age and sex-matched non-transgenic littermate mice were used as control for all mice experiments. Mice were randomly assigned in each group.
Blinding	The investigators were blinded to group allocation during data collection.

Reporting for specific materials, systems and methods

We require information from authors about some types of materials, experimental systems and methods used in many studies. Here, indicate whether each material, system or method listed is relevant to your study. If you are not sure if a list item applies to your research, read the appropriate section before selecting a response.

Materials & experimental systems

n/a	Involved in the study	
☐	☒	Antibodies
☐	☒	Eukaryotic cell lines
☒	☐	Palaeontology and archaeology
☐	☒	Animals and other organisms
☒	☐	Clinical data
☒	☐	Dual use research of concern

Methods

n/a	Involved in the study	
☒	☐	ChIP-seq
☐	☒	Flow cytometry
☒	☐	MRI-based neuroimaging

Antibodies

Antibodies used

Purified anti-GFP polyclonal antibody, Invitrogen #A11122
Pe-cy7 anti-CD11b monoclonal antibody, clone M1/70, Invitrogen # 25-0112-82
FITC anti-MHCII monoclonal antibody, clone M5/114.15.2, Invitrogen #11-5321-82
APC-eF780 anti-NK1.1 monoclonal antibody, clone PK136, Invitrogen #47-5941-82
PerCP-Cy5.5 anti-Foxp3 monoclonal antibody, clone FJK-16s, Invitrogen #15-5773-82
Purified anti-Collagen I polyclonal antibody, Biorad # 2150-1410
Purified anti-Collagen IV polyclonal antibody, Biorad # 2150-1470
Purified anti-GFP polyclonal antibody, Abcam #ab13970
Cy3 anti-aSMA monoclonal antibody, clone 1A4, Sigma #C6198
Purified anti-NG2 polyclonal antibody, Millipore #AB5320
APC anti-CD31 monoclonal antibody, clone MEC13.3, BD Bioscience, #17-0311-82
BV785 anti-CD31 monoclonal antibody, clone 390, BD OptiBuild, # 740879
Purified anti-CD3 monoclonal antibody, clone 500A2, BD Bioscience #14-0033-85
Alexa Fluor 700 anti-CD4 monoclonal antibody, clone RM4-5, BD Bioscience # 557956
V500 anti-CD45.2 monoclonal antibody, clone 104, BD Horizon #562129
BV605 anti-Ly6C monoclonal antibody, clone AL-21, BD Horizon #563011
PE-CF594 anti-SiglecF monoclonal antibody, clone E50-2440, BD Horizon #562757
Biotin anti-PDGFRa monoclonal antibody, clone APA5, Thermo Fisher #13-1401-82
Biotin anti-PDGFRb monoclonal antibody, clone APB5, Thermo Fisher #13-1402-82
APC anti-PDGFRa monoclonal antibody, clone APA5, eBioscience #17-1401-81
eFluor 710 anti-CD8b monoclonal antibody, clone eBioH35-17.2, eBioscience #46-0083-82
APC anti-F4/80 monoclonal antibody, clone BM8, eBioscience #17-4801-82
eFluor 660 anti-IFN gamma monoclonal antibody, clone XMG1.2, eBioscience #50-7311-82
eFluor 660 Isotype control IgG1 monoclonal antibody, clone eBio299Arm, eBioscience #50-4888-80
PE anti-ICAM1 monoclonal antibody, clone 3E2, BDPharmigen # 5553253
PerCP-Cy5.5 anti-Ly6G monoclonal antibody, clone 1A8, BDPharmigen # 560602
BV711 anti-CD3 monoclonal antibody, clone 17A2, Biolegend #100349
BV785 anti-CD11c monoclonal antibody, clone N418, Biolegend #117335
PE anti-CD206 monoclonal antibody, clone C068C2, Biolegend #141706
BV711 Streptavidin, Biolegend # 405241
PerCP-Cy5.5 Streptavidin, eBioscience # 45-4317-82
Purified anti-mouse Pdpn antibody, gift from A. Farr (University of Washington, Seattle).
Alexa Fluor 488 anti-chicken IgY, Thermo Fisher #A11039
Alexa Fluor 488 anti-rabbit IgG, Thermo Fisher #A21441
Cy3-AffiniPure F(ab')2 fragment anti-syrian hamster IgG, Jackson immune #107-166-142
Alexa Fluor 488 anti-hamster IgG, Thermo Fisher #A21110
Biotin anti-CD31 monoclonal antibody, clone MEC13.3, BDPharmigen # 553371
Alexa Fluor 647 anti-rat IgG, Thermo Fisher # A21247
FITC anti-CD45.2 monoclonal antibody, clone 104, BDBioscience # 553772
PE anti-CD11b monoclonal antibody, clone M1/70, BDBioscience # 561689
FITC anti-F4/80 monoclonal antibody, clone BM8, Biolegend # 123108
Alexa Fluor 647 anti-hamster IgG, clone A21451, Invitrogen
Polyclonal anti-Axl, R&D # AF854
Goat IgG control, R&D # AB-108-C
Polyclonal anti-Cleaved Caspase 3, Cell Signaling, # 9661
Polyclonal anti-Phospho-Axl, R&D, # AF2228
PE-Cy7 anti-DPPIV/CD26 monoclonal antibody, clone H194-112, Biolegend # 137810
Biotin anti-FDC monoclonal antibody, clone FDC-M1, BDPharmigen # 551320
PE anti-MadCAM-1 monoclonal antibody, clone MECA-367 , Biolegend # 120709
FITC anti-CD34 monoclonal antibody, clone RAM34, eBioscience # 48-0341
eFluor 660 anti-Ki67 monoclonal antibody, clone SolA15, Invitrogen # 50-5698
BV510 anti-Ly6C monoclonal antibody, clone HK1.4, Biolegend #128033
APC-Cy7 anti-PDPN monoclonal antibody, clone 8.1.1, Sony #1237090
Purified anti-CD8 antibody, Biolegend #100746
Rat IgG2a control, Biolegend #400544

Validation

All antibody used in this study were commercially available. Validation for FACS, immunofluorescence on frozen sections or in vivo studies is accessible from the supplier website (direct testing or by providing adequate references). We performed addditional antibody validation using isotype controls, streptavidins or secondary antibody only when necessary. We validated GFP staining as positive signal using GFP- tissues to set the negative threshold.

Eukaryotic cell lines

Policy information about cell lines and Sex and Gender in Research

Cell line source(s)

B16-MO5 (CVCL_WM77) were provided by Claude Leclerc (Institut Pasteur)

Authentication

B16-OVA (MO5) were previously validated (Fayolle et al., JI 1999). Cells were maintained in G418 (2 mg/ml) and hygromycin B (0,06 mg/ml) as described in methods.

Mycoplasma contamination

Cell lines were negative for mycoplasma and used within 4 passages.

Commonly misidentified lines (See ICLAC register)	No misidentified cell lines were used in this study

Animals and other research organisms

Policy information about studies involving animals; ARRIVE guidelines recommended for reporting animal research, and Sex and Gender in Research

Laboratory animals	ADAM12-GFP and ADAM12-DTR mice were previously described (Dulauroy et al, Nature Medicine 2012). TGFBR2floxed mice (Strain #012603) and TRAMP mice (Strain #003135) were obtained from Jackson Laboratory. Rip1Tag2 mice were obtained from the NCI Mouse Repository. We used mice from C57Bl/6 background, age 8-12w for MO5 studies, or as indicated in the text for TRAMP and RIPTag models.
Wild animals	No wild animals were used in this study
Reporting on sex	We used age and sex-matched males and females
Field-collected samples	None
Ethics oversight	Mice experiments were approved by the French Ministère de l'éducation nationale, de l'enseignement supérieur et de la recherche.

Note that full information on the approval of the study protocol must also be provided in the manuscript.

Flow Cytometry

Plots

Confirm that:

☒ The axis labels state the marker and fluorochrome used (e.g. CD4-FITC).

☒ The axis scales are clearly visible. Include numbers along axes only for bottom left plot of group (a 'group' is an analysis of identical markers).

☒ All plots are contour plots with outliers or pseudocolor plots.

☒ A numerical value for number of cells or percentage (with statistics) is provided.

Methodology

Sample preparation	Tumors were cut in small pieces and processed in a solution composed of DMEM (Gibco), Liberase TL (0,26 Wunit/mL; Roche) and DNase I (1 U/ml; ThermoFisher) for 30 minutes, with manual dissociation by pipetting every 10 minutes. Cells were filtered through a 100-µm and a 40µm mesh, washed and then processed for cell staining.
Instrument	Data were analyzed using BD LSRFortessa cytometer. Cells were sorted using FACSARIA III (BD Biosciences)
Software	Flow cytometry data were analyzed with Flowjo v.10 software.
Cell population abundance	Purity of the samples were determined in preliminary experiments by analyzing the post-sort population by FACS. Additional details are provided in the legends.
Gating strategy	For FACS experiments, we used FSC/SSC gates to eliminate debris and discriminate doublets, and excluded dead cells using DAPI (unfixed samples) or Live-dead (fixed samples). For further gating of specific cell populations, boundaries between positive and negative staining were determined using isotype controls, secondary antibody alone or streptavidins alone. For all FACS experiments of GFP+ cells, a GFP negative sample (originating from a GFP– littermate) was systematically used to define the positive/negative signal.<br>For cell sorting of stromal cells, we first excluded debris, dead cells, doublet cells, hematopoietic (CD45+) and endothelial (CD31+) cells, and then selected for positive stromal cell markers, as indicated in the legends/supp information. For isolation of GFP+ cells from tissues, the positive gate for GFP+ cells was determined using GFP– littermates with similar treatments.

☒ Tick this box to confirm that a figure exemplifying the gating strategy is provided in the Supplementary Information.
